# Role of tonic GABAergic currents during pre- and early postnatal rodent development

**DOI:** 10.3389/fncir.2013.00139

**Published:** 2013-09-03

**Authors:** Werner Kilb, Sergei Kirischuk, Heiko J. Luhmann

**Affiliations:** Institute of Physiology and Pathophysiology, University Medical Center of the Johannes Gutenberg UniversityMainz, Germany

**Keywords:** GABA_A_ receptor, GABA_B_ receptor, GABA transporter, GABA metabolism, neuronal proliferation, migration, taurine, review

## Abstract

In the last three decades it became evident that the GABAergic system plays an essential role for the development of the central nervous system, by influencing the proliferation of neuronal precursors, neuronal migration and differentiation, as well as by controlling early activity patterns and thus formation of neuronal networks. GABA controls neuronal development via depolarizing membrane responses upon activation of ionotropic GABA receptors. However, many of these effects occur before the onset of synaptic GABAergic activity and thus require the presence of extrasynaptic tonic currents in neuronal precursors and immature neurons. This review summarizes our current knowledge about the role of tonic GABAergic currents during early brain development. In this review we compare the temporal sequence of the expression and functional relevance of different GABA receptor subunits, GABA synthesizing enzymes and GABA transporters. We also refer to other possible endogenous agonists of GABA_A_ receptors. In addition, we describe functional consequences mediated by the GABAergic system during early developmental periods and discuss current models about the origin of extrasynaptic GABA and/or other endogenous GABAergic agonists during early developmental states. Finally, we present evidence that tonic GABAergic activity is also critically involved in the generation of physiological as well as pathophysiological activity patterns before and after the establishment of functional GABAergic synaptic connections.

The GABAergic system is critically involved in neuronal development (e.g., Ben-Ari, 2002; Ben-Ari et al., 2007; Wang and Kriegstein, 2009; Kilb, 2012), influencing virtually all developmental steps from neurogenesis (LoTurco et al., 1995) to the establishment of neuronal connectivity (Wang and Kriegstein, 2008). Since many of these events occur before the onset of synaptogenesis, a tonic, extrasynaptic GABAergic transmission may be important. In the following sections we will first describe the development of the GABAergic system, with special emphasize on all elements that support the particular role of extrasynaptic transmission. Subsequently, we will describe the influence of GABA on various developmental events and present evidence for a critical role of non-synaptic signaling in these processes. In addition, we will summarize observations that demonstrate an important role of extrasynaptic GABAergic transmission in the developing brain after the formation of GABAergic synapses and after onset of GABAergic synaptic transmission. And finally, we like to discuss the origin and nature of additional endogenous GABAergic agonists that mediate extrasynaptic effects during development.

Most studies mentioned in this review describe the development and influence of the GABAergic system during prenatal phases and the first postnatal week in rodents. This period is to some extent comparable to prenatal development in humans (Romijn et al., 1991), although a general comparison of pre- and perinatal stages between rodents and humans is complicated due the relatively advanced human brain development and the complex expansion pattern of different cortical areas during onto- and phylogenesis (Clancy et al., 2007; Hill et al., 2010). In addition, we like to emphasize that substantial developmental progress occurs during the first postnatal week in rodents and that at one given day of early development, neurons in different cortical regions and layers differ by 2–3 days in their developmental stage.

## DEVELOPMENT OF EXTRASYNAPTIC AND SYNAPTIC GABAergic TRANSMISSION

**FIGURE 1:**
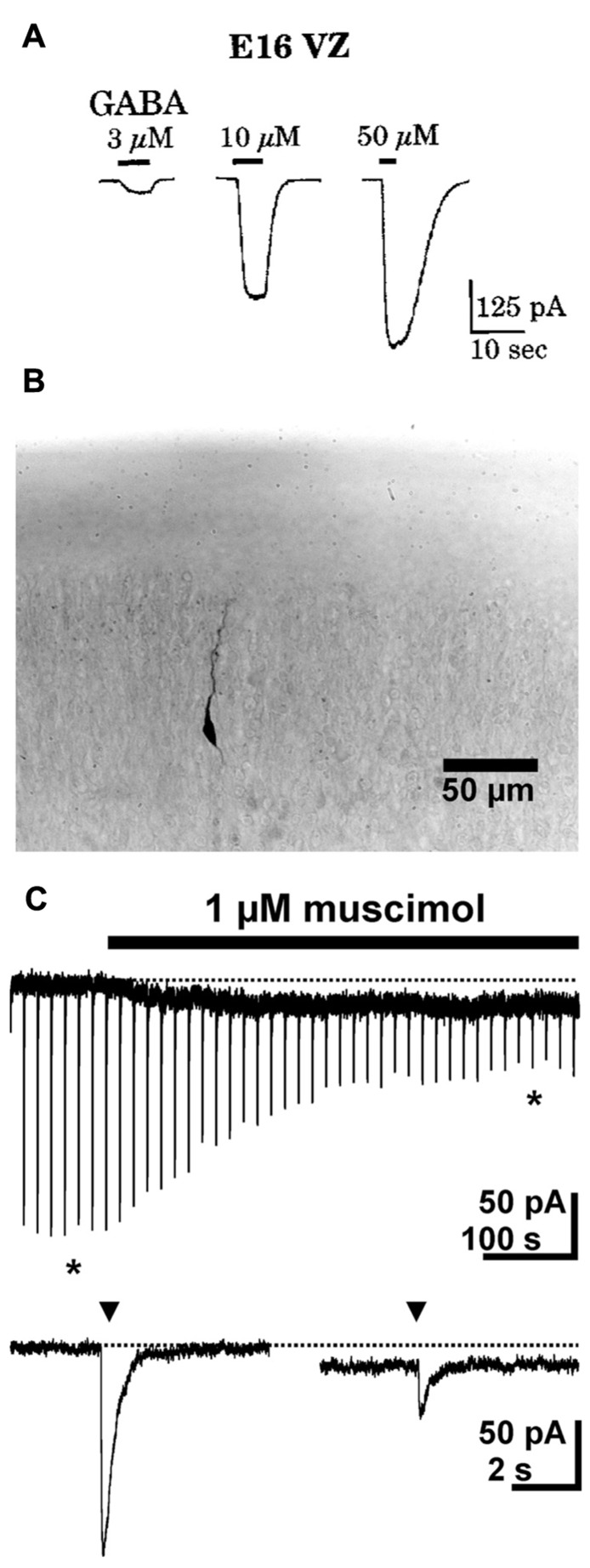
**GABAergic currents during early development.** **(A)** Whole-cell recordings from cells in ventricular zone of embryonic rats (E16) revealed dose-dependent GABAergic currents. **(B)** Photomicrograph of a biocytin-stained migrating neuron in a P1 rat. **(C)** Whole-cell recordings show that in migrating neurons a short (2–3 ms) application of 1 mM GABA (triangles) induced fast inward currents, while bath application of 1 μM muscimol induced a long lasting tonic current and led to a marked desensitization of phasic responses. The typical responses shown below the continuous trace are marked by asterisks. Pictures taken with kind permission from Owens et al. (1999) **(A)** and Heck et al. (2007) **(B,C)**.

Cells in the developing nervous system respond to GABA at surprisingly early stages. The first evidence for this has been found in dissociated cells from the earliest phases of neurogenesis in the turtle brain, which show bicuculline sensitive responses upon GABA application (Shen et al., 1988). In rodents neuronal progenitors in the ventricular zone at embryonic day (E) 15 (LoTurco et al., 1995; Owens et al., 1999) as well as postmitotic migrating neurons (Heck et al., 2007) already reliably show GABAergic responses (**Figure 1**). In accordance with this early onset of GABAergic responses, the expression of GABA_A_ receptors also starts during very early brain development.

GABA_A_ receptors are heteropentameric molecules composed of 19 possible subunits (α1–6, β1–3, γ1–3, δ, ε, φ, π, and ρ–3). The subunit composition determines GABA affinity, channel conductance and kinetics, pharmacology and subcellular localization of the receptors (Farrant and Kaila, 2007). A few functional consequences of subtype compositions relevant for this review are (i) in particular the γ2 subunit determines the synaptic allocation of GABA_A_ receptors, while δ subunit containing receptors are exclusively located at extrasynaptic sites, (ii) α4–6 and ρ subunit containing receptors have been found predominantly at extrasynaptic localizations, while α1–3 containing receptors are supposed to constitute the synaptic GABA_A_ receptors, and (iii) δ subunit and ρ subunit containing receptors typically reveal a high GABA affinity and a slow and incomplete desensitization, appropriate for a tonic activation by interstitial GABA (reviewed in Farrant and Nusser, 2005; Farrant and Kaila, 2007).

*In situ* hybridization experiments in the neocortex revealed expression of GABA_A_ receptors as early as at E13 with the appearance of β3 subunits in the neuroepithelium (Araki et al., 1992). At E14/E15 α3, α4 are expressed in the developing cortical layers (Araki et al., 1992; Laurie et al., 1992). Between E15 and E17 γ2 subunit mRNA is detected in the neocortex, with the highest expression levels in the cortical plate (CP; Araki et al., 1992; Laurie et al., 1992; Van Eden et al., 1995). At E17 there is evidence that even α6 subunits, which are in the adult brain nearly exclusively located in the cerebellum (Luddens et al., 1990), are expressed in the cortical neuroepithelium (Poulter et al., 1992). In contrast, the α1 subunits characteristic for many mature GABA_A_ receptors are expressed relatively late between E19 and P0 (Poulter et al., 1992; Van Eden et al., 1995), while δ subunit expression is observed only postnatally (Laurie et al., 1992). These observations are supported by northern blot analyses which reveal expression of α2 and α4 at E18 in total brain homogenates, while α1 expression starts only after birth (MacLennan et al., 1991). On the other hand, for precursors of GABAergic interneurons traveling from the lateral ganglionic eminence to the cerebral cortex a stringent up-regulation of α1 and γ1–3 subunits occurs after they enter the cortex, which is directly linked to an increase of GABA affinity (Carlson and Yeh, 2011). To our knowledge no study has been published for rodents that investigated the prenatal appearance of different GABA_A_ receptor subunits on protein level. However, in rodents at the day of birth (P0) an intense α2 receptor immunoreactivity has been observed in the neocortex, while α1 receptors immunoreactivity is low, but detectable (Fritschy et al., 1994). In the primate neocortex a significant expression of α2, α4, and α5 was observed during prenatal development (Hornung and Fritschy, 1996; Huntsman et al., 1999), while α1 subunits appear shortly before birth and are substantially up-regulated in the first postnatal year (Hornung and Fritschy, 1996).

In the rodent hippocampus expression of mRNA for α2 and α5, but also γ2 subunits start at E15, while δ subunit mRNA was detected only after birth (Killisch et al., 1991; Laurie et al., 1992; Poulter et al., 1992). At E19 it has been found that neuroepithelial cells or early postmitotic cells in the hippocampus express predominantly α4 and α5 containing GABA_A_ receptors (Maric et al., 1999). Expression of α1 subunit mRNA appear only postnatally (Laurie et al., 1992; Poulter et al., 1992). Immunohistochemical studies in the perinatal hippocampus revealed a nearly absence of α1 subunits, while α2 subunits were highly abundant (Fritschy et al., 1994).

**FIGURE 2:**
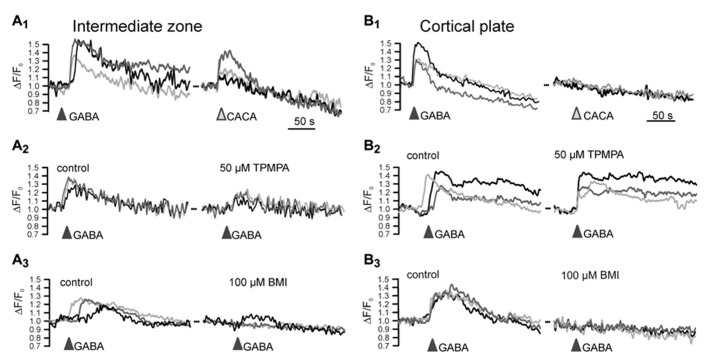
**Functional expression of ρ subunit containing GABA_A_ receptors in the intermediate zone.** Microfluorimetric registration of Ca^2+^ transients induced by application of 100 μM GABA and 100 μM of the ρ subunit specific agonist cis-4-aminocrotonic acid (CACA) in neurons from the intermediate zone **(A)** and cortical plate **(B)**. Note that CACA induced Ca^2+^ transients occurred only in the intermediate zone **(A_1_,B_1_)** that the ρ subunit containing GABA_A_ receptor antagonist (1,2,5,6-tetrahydropyridin-4-yl)met-hylphosphinic acid (TPMPA) was inefficient in the cortical plate **(A_2_,B_2_)** and that the GABA_A_ antagonist bicuculline (BMI) completely abolished GABAergic Ca^2+^ transients in the CP **(A_3_,B_3_)**. Modified from Denter et al. (2010).

Ionotropic GABA receptors constituted of ρ subunits (also termed GABA_C_ receptors) have a high GABA affinity, slow activation and inactivation kinetics and show little desensitization (Bormann, 2000). In accordance with these properties, they can mediate extrasynaptic GABAergic effects (Alakuijala et al., 2006). Expression of ρ subunits has been found in lower neocortical layers of the E15 mouse brain (Fukui et al., 2008) and in the early postnatal hippocampus (Rozzo et al., 2002). In accordance with the early expression of ρ subunits only in lower neocortical layers, functional ρ subunit containing GABA_A_ receptors are detected in the intermediate zone (IZ), while they are not expressed in the CP (Denter et al., 2010; **Figure 2**). The pharmacological properties of these receptors indicate that they most probably are ρ subunits containing heteropentamers, as has been also suggested for interneurons in the adult hippocampus and juvenile CA1 pyramidal neurons (Semyanov and Kullmann, 2002; Hartmann et al., 2004).

In summary, these studies demonstrate that in the cerebral cortex and hippocampus classical GABA_A_ and ρ subunit containing GABA receptors are expressed at early developmental stages, and that these receptors probably contain α2–α5 and ρ, but also γ2 subunits. Although the expression of γ2 subunits typically corresponds to a postsynaptic localization of GABA_A_ receptors, the relatively high expression levels of α4/α5 and ρ subunits are compatible with extrasynaptic GABA_A_ receptors. In contrast, there is compelling evidence that δ subunits, which are typical for classical extrasynaptic receptors in the immature brain (Farrant and Nusser, 2005), are lacking during early embryonic development.

During embryonic and early postnatal development ionotropic GABA receptors mediate in pyramidal neurons depolarizing membrane responses (Mueller et al., 1984; Ben-Ari et al., 1989; Owens et al., 1996; Lamsa et al., 2000; Achilles et al., 2007; Valeeva et al., 2013). These depolarizing GABAergic responses are caused by Cl^-^ efflux via GABA_A_ receptors due to the high intracellular Cl^-^ concentration in developing neurons (Ben-Ari et al., 2012), and play an essential role for the trophic actions of GABA during early development (Ben-Ari, 2002; Represa and Ben-Ari, 2005; Wang and Kriegstein, 2009; Kilb et al., 2011; but see Cancedda et al., 2007). In GABAergic interneurons GABA_A_ receptor activation mediate comparable, slightly depolarizing actions in interneurons during early postnatal stages as well as in the adult brain (Banke and McBain, 2006).

In addition to ionotropic GABA receptors, metabotropic GABA_B_ receptors are also important elements of the immature GABAergic system. Functional GABA_B_ receptors are heterodimers consisting of GABA_B_1R and GABA_B_2R subunits and are mainly located distant to release sites, suggesting an extrasynaptic activation (Ulrich and Bettler, 2007). A co-localized protein expression of both GABA_B_ receptor subunits, and thus presumably also functional GABA_B_ receptors, has been found in hippocampal and cortical regions after E15 (Behar et al., 2001; López-Bendito et al., 2002; Li et al., 2004). Accordingly, evidence for a functional implication of GABA_B_ receptors on different developmental events has been demonstrated in the immature neocortex and hippocampus (Behar et al., 2001; López-Bendito et al., 2003; Kirmse and Kirischuk, 2006; McClellan et al., 2007). In the first postnatal week a reliable presynaptic effect of GABA_B_ receptors is observed in the rodent neocortex and hippocampus, while a postsynaptic current is less developed (Luhmann and Prince, 1991; Fukuda et al., 1993; Gaiarsa et al., 1995).

A peculiar observation of the *in situ* hybridization studies for GABA_A_ receptor subunits was the early expression of γ2 subunits, which are implicated in synaptic clustering of GABA_A_ receptors, during early embryonic stages. However, γ2 subunits mediate the postsynaptic clustering of GABA_A_ receptors via an interaction with the scaffolding proteins gephyrin and collybistin (Kneussel and Betz, 2000; Mukherjee et al., 2011). While gephyrin is expressed at high levels in the neuroepithelium already at E14, collybistin expression was observed in the cortical anlage only in regions with postmitotic neurons, which strongly suggests that GABA_A_ receptors cannot form postsynaptic clusters in the ventricular and subventricular zones and on migrating neurons (Kirsch et al., 1993; Kneussel et al., 2001).

GABA required for the activation of these GABA receptors can originate either from GABAergic neurons or GABAergic fibers. GABAergic interneurons are generated in rodents mainly in the medial and caudal ganglionic eminence and migrate tangentially to the neocortex and hippocampus (Anderson et al., 1997; Pleasure et al., 2000; Miyoshi et al., 2010), where they, depending on their origin, differentiate in the diverse types of GABAergic interneurons (Tricoire et al., 2011). In the human neocortex a substantial fraction of the GABAergic interneurons is, however, generated in the ventricular zone of the dorsal pallium (Petanjek et al., 2008; Yu and Zecevic, 2011). The first GABA positive neurons are detectable in the primordial plexiform layer of the cortical anlage already at E12 (Del Rio et al., 1992). In subsequent embryonic stages GABAergic neurons are abundant in all layers of the developing cortex between the marginal zone and the subventricular zone (Lauder et al., 1986; Van Eden et al., 1989; Cobas et al., 1991; Del Rio et al., 1992). In the human neocortex the first GABAergic neurons were detected at gestational week 6.5 even before the appearance of the CP (Zecevic and Milosevic, 1997). In the rodent hippocampus GABAergic interneurons were first detected between E14 and E16 in the inner marginal zone, the subplate and the subventricular zone (Soriano et al., 1994; Kanold and Luhmann, 2010). In addition to this early appearance of GABAergic neurons, a dense network of GABAergic fibers originating from extracortical regions reach the cortical anlage during these early embryonal stages even before the appearance of GABAergic neurons (Lauder et al., 1986; Del Rio et al., 1992; Ma and Barker, 1995).

GABA is mainly produced by the glutamic acid decarboxylases (GAD), which is expressed in two major isoforms. GAD-65 is considered to mediate the production of GABA intended for synaptic release, while GAD-67 is supposed to maintain cytoplasmic GABA levels (Soghomonian and Martin, 1998). In the rodent neocortex expression of GAD-67 is detectable at E15 (Ma and Barker, 1995) in the germinal zone of the cortical anlage, while GAD-65 occurs delayed and is observable only after P6 (Kiser et al., 1998). In the hippocampus both GAD-65 and GAD-67 protein could be detected at E18, with both isoforms expressed mainly at somatic locations during prenatal development (Dupuy and Houser, 1996). Expression of GAD has also been found in fetal human brain before gestational week 15 (Das and Ray, 1997), when GAD-67 is the prominent isoform (Chan et al., 1997). Thus in particular a somatic generation of GABA can appear during early stages of corticogenesis. In contrast, the GABA degrading enzymes GABA transaminase and succinate semialdehyde dehydrogenase reveal low expression levels in the pre- and early postnatal rodent neocortex (Pitts and Quick, 1967; Kristt and Waldman, 1986), suggesting that a substantial portion of GABA is distributed within the CNS via the interstitium.

For synaptic release GABA must be accumulated in transmitter vesicles by the vesicular inhibitory amino acid transporter (vIAAT) or vesicular GABA transporter (vGAT; Wojcik et al., 2006). At P0 the expression of vGAT in the neocortex is rather low and mostly restricted to fibers (Minelli et al., 2003). In the rodent hippocampus GABAergic inputs were observed before birth in about 75% of pyramidal neurons at P0 and in 65% of interneurons (Tyzio et al., 1999; Hennou et al., 2002), indicating the early appearance of functional GABAergic synapses, but also that synaptic spillover may be a source of extrasynaptic GABA receptor activation. No GABAergic synaptic responses were observed in proliferative regions of the cerebral cortex (Owens et al., 1999). On the other hand, reliable tonic GABAergic currents were found in the embryonic neocortical neurons in the ventricular zone (LoTurco et al., 1995) and the CP (Owens et al., 1999), and in neuronal cultures of embryonic hippocampal neurons (Valeyev et al., 1998). These observations indicate that GABAergic responses precede synaptic GABAergic transmission, strongly suggesting that non-synaptic transmission plays an important role during prenatal development. Indeed, there is compelling evidence that during these stages a substantial part of basal, but also of stimulated GABAergic transmission occur via non-vesicular release (Demarque et al., 2002). The tonic GABAergic currents observed in neuronal cultures of embryonic hippocampal neurons suggest that hippocampal neurons itself can be a source of GABA in these cells (Valeyev et al., 1998). Subsequent *in vitro* experiments revealed that isolated neurons from the CP can release GABA in sufficient large amounts to obtain micromolar concentrations in the supernatant (Behar et al., 2001). In immature neocortical slices an extracellular GABA concentration of 250–500 nM, and thus sufficiently high to activate high affinity ionotropic GABA receptors or GABA_B_ receptors has been found (Cuzon et al., 2006; Dvorzhak et al., 2010).

Possible candidates for a non-vesicular GABA release are GABA transporters (GATs). In the adult nervous system these transporters mediate the uptake of GABA from interstitial space, show mainly a neuronal localization of the GAT-1 isoform and mainly glial localization of GAT-3 isoform (Borden, 1996). Accordingly these two subtypes are responsible for controlling extracellular GABA from vesicular and non-vesicular sources, respectively (Song et al., 2013). However, these transporters can also act in reverse mode and thus release GABA from cells (Richerson and Wu, 2003; Kirischuk and Kilb, 2012). In particular the high intracellular Cl^-^ concentration in immature neurons, which directly influences GAT mediated GABA transport by determining the reversal potential of this Cl^-^ dependent transmembrane transporter, can result in less efficient uptake or even a reversal of the transport mode (Kirischuk and Kilb, 2012). In the mouse brain GAT-1 expression starts at E14 in the medial ganglionic eminence and is detected at E16 in the neocortical subventricular zone (Evans et al., 1996). GAT-3 is already expressed in the neocortical subventricular zone at E14 (Evans et al., 1996). In contrast, in the developing hippocampus GAT-1 expression is low at early postnatal age, with GAT-3 expression dominating (Evans et al., 1996). Interestingly, between the end of the first postnatal week and the end of the first postnatal month a transient somatic location of GAT-1 expression has been demonstrated in the rat neocortex and hippocampus (Yan et al., 1997). In addition, in the immature rat cortex GAT-1 is abundant in astrocytes and GAT-3 in neurons (Yan et al., 1997; Minelli et al., 2003), despite the mainly neuronal localization of GAT-1 and the mainly glial localization of GAT-3 in the adult CNS (Borden, 1996; Conti et al., 2004). Both observations suggest a substantial shift in the functional role of GAT during development. A direct implication of GAT-1 for GABA release has been demonstrated in tangentially migrating precursors of GABAergic interneurons (Poluch and Konig, 2002), where glutamatergic activation leads to a non-vesicular GABA release by a Na^+^-increase driven reversal of the GAT-1 transporter (Pin and Bockaert, 1989). On the other hand, in cortical neuroblasts the activity dependent GABA release does not depend on GATs (Liu et al., 2005), indicating that additional, currently unknown pathways may contribute to non-vesicular GABA release during early development. And finally, it has been shown for the early postnatal hippocampus and neocortex that GAT-1 is already effectively removing GABA from the extracellular space and thereby directly regulates tonic GABAergic currents and affects intrinsic neuronal activity (Sipila et al., 2004, 2007; Bragina et al., 2008).

Overall, these studies suggest that in the immature neocortex and hippocampus all essential elements for functional GABAergic transmission appear at very early stages, while the elements for reliable synaptic GABA release and GABA_A_ receptor clustering occur at later developmental stages. In summary, these findings indicate that activation of extrasynaptic GABAergic receptors underlies the diverse trophic actions of GABA during early neuronal development.

## INFLUENCE OF EXTRASYNAPTIC GABAergic TRANSMISSION ON CRITICAL EVENTS DURING PRE- AND EARLY POSTNATAL DEVELOPMENT

GABA has been considered as a major neurotrophic factor during embryonic development (Varju et al., 2001; Owens and Kriegstein, 2002; Represa and Ben-Ari, 2005; Wang and Kriegstein, 2009). Different developmental events, ranging from proliferation to the establishment of mature synaptic circuits depend on GABA, with a substantial portion of these events occurring without a major contribution of synaptic processes.

Application of GABAergic agonists increases DNA synthesis and the proliferation of neuroblasts in the ventricular zone, whereas it decreases proliferation in the subventricular zone (LoTurco et al., 1995; Haydar et al., 2000). Most probably the majority of these neuronal progenitors in the ventricular zone are radial glial cells (Noctor et al., 2001), on which functional GABA_A_ receptors have been reported (Noctor et al., 2002). While alterations in proliferation induced by the external GABA application demonstrate that GABA has the potential to directly interfere with neurogenesis, the observation that inhibition of GABA_A_ receptors induces the opposite effect clearly shows that endogenously released GABA regulates neurogenesis in ventricular and subventricular zones (LoTurco et al., 1995; Haydar et al., 2000). In addition to GABA_A_ receptors, it has been also shown that GABA_B_ receptors are involved in controlling neurogenesis (Fukui et al., 2008) and gliogenesis (Luyt et al., 2007). It can be assumed that extrasynaptic GABAergic transmission influences these processes, since in both layers synaptic GABA release has not been reported so far. This mechanism has been documented in adult neurogenesis, where GABAergic neuroblasts in the subventricular zone release GABA via non-vesicular mechanisms, which in turn impede the proliferative activity of glia-derived progenitor cells (Liu et al., 2005). In addition, it could be shown that during adult neurogenesis α4 subunit containing GABA receptors are involved in the regulation of neurogenesis (Duveau et al., 2011), which also suggests that extrasynaptic transmission is essential. Neurogenesis in the neocortex is also impeded by glutamate, and this glutamatergic inhibition of proliferation of glutamatergic pyramidal neurons is supposed to serve as a feedback mechanism to control the number of excitatory neurons (Wang and Kriegstein, 2009). However, because in rodents the ventricular and subventricular zones of the cortical anlage do not give rise to GABAergic interneurons, the functional role of GABAergic control of proliferation is probably more complicated.

In the mammalian brain the neurotransmitter phenotype of neurons is determined by different transcription factors and is normally established with neurogenesis (Ma, 2006). The observation that granule cells of the dentate gyrus can switch their neurotransmitter phenotype in an activity dependent manner (Romo-Parra et al., 2003), suggest that during neuronal development the expression of transcription factors may also be affected by neurotransmitters and their receptors. In the central nervous system of *Xenopus* it has already been shown that GABA can directly influence neurotransmitter specification via GABA_B_ receptors (Root et al., 2008), however, for mammalian species it has not been demonstrated yet whether GABA receptors can influence the neurotransmitter phenotype.

**FIGURE 3:**
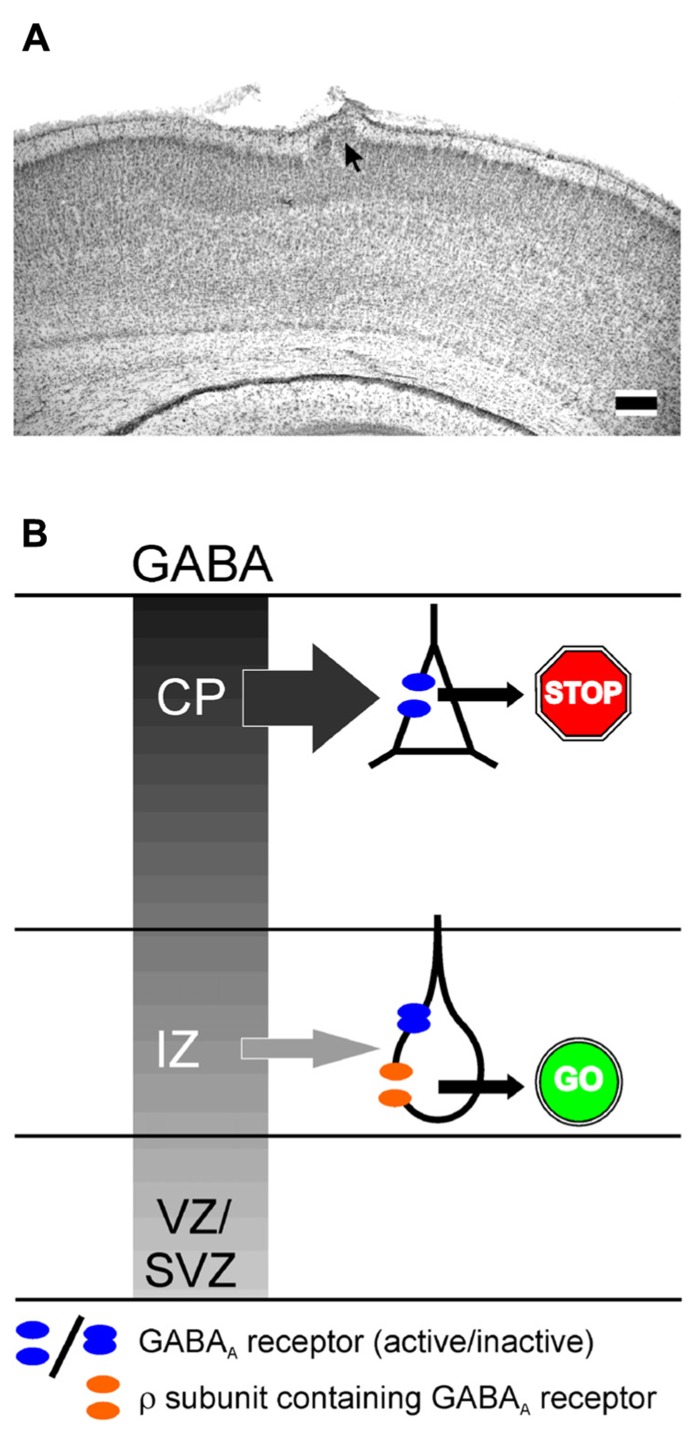
**Influence of GABA_A_ receptors on migration.** **(A)** Migration defect induced by GABA_A_ receptor inhibition *in vivo*. Digital photograph of 50-μm-thick Nissl-stained coronal sections showing a heterotopia (arrow) of a P7 rat treated with a BMI loaded Elvax implant at P0. Scale bars correspond to 200 μm. **(B)** Schematic drawing illustration the effect of classical and ρ subunit containing GABA_A_ receptors on radial migration in the developing neocortex. The gray gradient represents the outside directed GABA gradient. In the intermediate zone (IZ) migrating neurons express classical GABA_A_ and ρ subunit containing GABA_A_ receptors. In the cortical plate (CP) only classical GABA_A_ receptors are found. Due to the outside directed GABA gradient the low-affinity classical GABA_A_ receptors are only activated in the CP, where they contribute to termination of migration (STOP sign). The lower GABA concentration in the IZ is only sufficient to activate the high affinity ρ subunit containing GABA_A_ receptors, which is necessary to support migration in the IZ (GO sign). Modified from Heck et al. (2007) **(A)** and Denter et al. (2010) **(B)**.

A variety of *in vitro* and *in vivo* studies reported that GABA is an important determinant of neuronal migration, acting as chemoattractant, regulating cell mobility and influencing initiation and termination of the migration process (Behar et al., 1996; Heng et al., 2007; Manent and Represa, 2007). A selective inhibition of GABA_A_ receptors enhances radial migration in neocortical organotypic slice cultures (Behar et al., 2000; Heck et al., 2007) and *in vivo* induces severe cortical malformations leading to upper cortical heterotopia (Heck et al., 2007), suggesting that GABA_A_ receptors provide a stop signal for migrating neurons (**Figure 3A**). In contrast, specific inhibition of ρ subunit containing GABA_A_ receptors or a simultaneous inhibition of both subclasses of GABA_A_ receptors impedes radial migration (Behar et al., 2000; Denter et al., 2010), suggesting that ρ subunit containing GABA_A_ receptors support the migration out of deeper cortical layers (**Figure 3B**). Functional ρ subunit containing GABA_A_ receptors are only found in the subcortical regions of the developing cortex that lack evidence for synaptic release (Denter et al., 2010), indicating that they are predominantly activated by non-synaptically released GABA. Since so far no synaptic inputs on migrating neurons have been demonstrated, it has been suggested that the stop signal for migrating neurons is mediated by the higher interstitial GABA concentrations in superficial layers of the developing cortex (Behar et al., 2000). Despite the different effects of both subclasses of GABA_A_ receptors on radial migration, the effect of ionotropic GABA receptors on migration is most probably mediated by depolarization-induced Ca^2+^ transients, which are essential for the induction and maintenance of radial migration (Komuro and Rakic, 1996, but see Cancedda et al., 2007). In addition, it has been demonstrated that GABA facilitates the tangential migration of GABAergic interneurons to the appropriate neocortical locations via GABA_A_ receptors (Cuzon et al., 2006; Inada et al., 2011). Metabotropic GABA_B_ receptors stimulate the transition of radially migrating neurons from the IZ to the CP (Behar et al., 2001) and mediate the migration of interneurons to their appropriate locations in the neocortex during embryonic stages (López-Bendito et al., 2003). In the immature hippocampus GABA_A_ receptors promote radial migration (Manent et al., 2005). Importantly, neuronal migration is unaffected in animals in which the synaptic release is completely suppressed (Manent et al., 2005), again emphasizing the essential role of non-synaptically released GABA acting on extrasynaptic receptors for neuronal migration in the hippocampus.

GABA also exerts a direct effect on neurite growth and axon elongation (see Sernagor et al., 2010 for review). A variety of studies demonstrated that GABA application promotes the outgrowth and ramification of dendrites in neocortical and hippocampal neurons (e.g., Barbin et al., 1993; Maric et al., 2001). This dendrite promoting effect of GABA relies on depolarizing GABAergic responses and subsequent Ca^2^^+^ signals (Maric et al., 2001; Cancedda et al., 2007; Wang and Kriegstein, 2008). Dendritic ramification is inhibited by GABAergic antagonists (Maric et al., 2001), indicating that endogenous GABA is required for normal neurite formation. The cell culture studies of Maric et al. (2001) convincingly showed that not only blocking GABA_A_ receptors, but also inhibition of GAD severely impairs dendrite formation, which strongly suggests that an autocrine release of GABA acting on extrasynaptic GABA_A_ receptors is required for this effect. In cell cultures from embryonic cortical neurons axonal growth is also accelerated by GABA_A_ receptor activation via Ca^2^^+^ and calmodulin-dependent kinase 1 activation (Ageta-Ishihara et al., 2009). Since this effect is reversed by GABA_A_ receptor antagonists and axon extension partly occurs before the appearance of synaptic GABA release, it has been suggested that tonic GABAergic effects also contribute to the facilitating GABA effect on axon extension (Sernagor et al., 2010). Activation of presynaptic GABA_B_ receptors is required to stabilize developing GABAergic synapses of basket cells in the mouse occipital neocortex (Fu et al., 2012). On the other hand, it has been shown that already in the immature hippocampus and neocortex tonic activation of presynaptic GABA_B_ receptors reduces GABA release (Safiulina and Cherubini, 2009; Dvorzhak et al., 2010).

Subsequent developmental events are also directly influenced by GABAergic signaling. For example it has been shown that depolarizing GABAergic responses are essential for synaptogenesis (Wang and Kriegstein, 2008). But because these events occur mostly after the onset of synaptogenesis and after the functional expression of GABAergic synaptic inputs (e.g., Owens et al., 1999; Tyzio et al., 1999; Hennou et al., 2002; Kilb et al., 2004), synaptic GABAergic effects may dominate these events. However, it has been shown that this GABAergic influence on synaptogenesis is closely linked to *N*-methyl-D-aspartate (NMDA) receptors (Wang and Kriegstein, 2008). In immature neurons the Mg^2+^ block from NMDA receptors is released by an GABAergic depolarization before 2-amino-3-(3-hydroxy-5-methyl-isoxazol-4-yl)propanoic acid (AMPA) mediated synaptic inputs appear (Leinekugel et al., 1997), leading to spontaneous correlated network activity, which is typical for the developing brain (Ben-Ari et al., 1989; Garaschuk et al., 1998; Dupont et al., 2006) and which probably plays an essential role for the maturation of neuronal networks (see Khazipov and Luhmann, 2006; Ben-Ari et al., 2007; Kilb et al., 2011 for review). But since tonic GABAergic currents are essential to drive such events in the immature hippocampus (Sipila et al., 2005), it can be assumed that tonic GABAergic currents continue to contribute to the trophic GABA action even after the onset of GABAergic synaptic transmission.

In summary, these studies provide convincing evidence that tonic GABAergic currents control the genesis, migration and differentiation of neurons during early development. All these events occur either before the onset of synaptic GABAergic transmission or in neurons that do not receive synaptic inputs, indicating that extrasynaptic GABAergic signaling is essential for these processes. Beside this correlative indication, the studies by Maric et al. (2001); Liu et al. (2005), and Manent et al. (2005) provide strong evidence for the important role of non-synaptically released GABA on neurogenesis, migration, and differentiation. An additional evidence for the dominating role of non-vesicular released GABA on cortical development is the observation that no anatomical abnormalities are detected in the neocortex of vGAT knockout mice, in which synaptic GABA release is absent (Wojcik et al., 2006). Similarly, a normal neocortical appearance, dendritic arborization and even synaptic structure has been described in perinatal mice after complete suppression of synaptic release in munc-18 knockout mice (Verhage et al., 2000; Demarque et al., 2002), again emphasizing the role of non-vesicular release and extrasynaptic receptors for early neuronal development.

## TONIC CURRENTS REGULATE EXCITATION AFTER GABAergic SYNAPTOGENESIS

Tonic GABAergic currents also strongly influence the activity of the immature nervous system after the onset of synaptic activity. As discussed above, in particular the different subunit composition of GABA_A_ receptors or distinct distribution and transport modes of GATs can contribute to these age-dependent effects. For instance in the rodent hippocampus α_5_ subunit expression (Ramos et al., 2004) and, correspondingly, tonic GABAergic currents (Holter et al., 2010) are up-regulated during the first postnatal week. Due to the depolarizing GABAergic responses during these stages (Ben-Ari et al., 2012), these tonic GABAergic currents increase the excitability of the immature hippocampus (although GABAergic responses can mediate shunting inhibition even at depolarizing potentials; Kolbaev et al., 2011). Accordingly, early network oscillations in the hippocampus, which depend on glutamatergic transmission and membrane properties of intrinsically bursting pyramidal neurons (Ben-Ari et al., 1989; Bolea et al., 1999; Sipila et al., 2006), are driven by a tonic GABAergic depolarization of these cells (Sipila et al., 2005). In the early postnatal hippocampus tonic GABAergic currents, mediated by α5 and γ2 subunit containing GABA receptors, enhance the excitability of pyramidal neurons, but not of interneurons (Marchionni et al., 2007). Interestingly, in the adult hippocampus moderate tonic currents are more prominent in interneurons and can even promote excitation in these cells (Semyanov et al., 2003), although GABA_A_ receptor activation mediate similar, slightly depolarizing actions in interneurons at both developmental stages (Banke and McBain, 2006). Due to this slight depolarizing action, moderate tonic currents mediate an excitatory action in mature hippocampal interneurons, while shunting inhibition dominates at larger tonic conductances a (Song et al., 2011).

**FIGURE 4:**
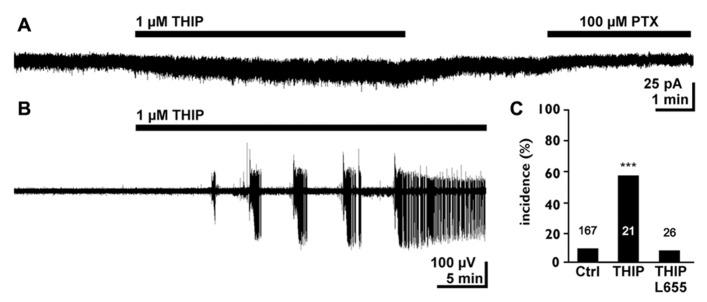
**Tonic GABAergic currents can promote epileptiform activity in the early postnatal hippocampus.** **(A)** Whole cell recording of a CA3 pyramidal neuron in the P4–7 hippocampus. The experiment was performed in the continuous presence of 40 μM dl-2-amino-5-phosphopentanoic acid (APV), 10 μM 6-cyano-7-nitroquinoxaline-2,3-dione (CNQX), and 1 μM gabazine (GBZ) to block synaptic components. Note that bath application of 1 μM 4,5,6,7-tetrahydroisooxazolo[5,4-c]pyridin-3-ol hydrochloride (THIP) induced a small tonic current and that the GABA_A_ antagonist picrotoxin uncovers a tonic GABAergic component. **(B)** Field potential recording illustrating that bath application of 1 μMTHIP in low-Mg^2+^ solution induced epileptiform discharges. **(C)** Statistical analysis demonstrating that 1 μMTHIP significantly increased the incidence of epileptiform discharges (compared to the control condition in low-Mg^2+^ solution). This proconvulsive THIP effect was prevented in the presence of the α5 selective antagonist L-655,708. Modified from Kolbaev et al. (2012).

In the immature hippocampus a moderate increase in tonic GABAergic currents mediated by α5 containing receptors promote epileptiform discharges under low-Mg^2+^ condition, which are insufficient to induce epileptiform discharges in this preparation (Kolbaev et al., 2012; **Figure 4**). In the hippocampus of early postnatal rats a tonic activation of GABA_B_ receptors does not control basal or stimulated GABA release (Caillard et al., 1998), although the GABA_B_ specific agonist Baclofen reduces the amplitude of GABAergic postsynaptic currents, indicating the functional expression of GABA_B_ receptors in this structure (Caillard et al., 1998). In contrast, it has been shown that a tonic activation of GABA_B_ receptors decreases neurotransmitter release in GABAergic synapses in early postnatal hippocampus and neocortex (Safiulina and Cherubini, 2009; Dvorzhak et al., 2010). This observation indicates that ambient GABA can mediate a stringent feedback control via GABA_B_ receptors during a developing stage when GABA can generate excitatory responses (Ben-Ari, 2002; Valeeva et al., 2010). In addition, these presynaptic GABA_B_ receptors may also limit the amount of ambient GABA originating from synaptic release. However, in summary these results demonstrate that a tonic activation of GABA_A_ receptors can increase the excitability in early postnatal circuits.

## ORIGIN AND NATURE OF ENDOGENOUS GABAergic AGONISTS

The important role of GABA for neuronal development was challenged by the observation that even a complete knockout of both GAD-65 and GAD-67 did not induce gross disturbances in the neocortex and hippocampus until P0, albeit a virtually absence of GABA in the brains of these animals (Ji et al., 1999). Although this study may refute the importance of GABA as neurotrophic substance during prenatal development, it can also indicate that other factors can compensate the lack of GABA or even represent an additional important trophic neurotransmitter during early corticogenesis.

In this respect it is important to reconsider that most studies investigating tonic currents identify such current by the blockade of GABA_A_ receptors and thus cannot provide any information about the nature of the endogenous ligand of extrasynaptic GABA receptors. One intriguing candidate for such a substance is taurine, which is an agonist of GABA_A_, GABA_B_, and glycine receptors (Albrecht and Schousboe, 2005). In rat and human fetal brain taurine is the most abundant neurotransmitter (Das and Ray, 1997; Benitez-Diaz et al., 2003). In early postnatal cortex a glycinergic agonist, presumably taurine, is released upon electrical stimulation in a Ca^2+^ and action potential independent manner (Flint et al., 1998) and in the presence of a hypoosmolar solution (Flint et al., 1998; Kilb et al., 2008), indicating that taurine can be release in the immature central nervous system (CNS) mainly by non-synaptic processes. Possible release pathways are volume-sensitive organic osmolyte channels or a reversal of the taurine transporter (Ando et al., 2012). Analysis of the chemoattractant diffusible factors released by the CP neurons also identified taurine as a possible candidate (Behar et al., 2001) and it has been shown by the same authors that taurine modulates radial migration via activation of GABA_B_ receptors (Behar et al., 2001). In accordance with these *in vitro* studies, migration deficits have been found in kittens born from taurine-deficient mothers (Palackal et al., 1986). Taurine may, however, act mainly as an agonist for glycine receptors, which have been found in the immature CNS (Wu et al., 1992; Flint et al., 1998; Kilb et al., 2002; Okabe et al., 2004); which are supposed to be mainly activated by non-synaptically released taurine (Mori et al., 2002) and which are also directly involved in early developmental events like migration (Nimmervoll et al., 2011).

However, the exact concentrations of interstitial GABA and taurine are unknown, since most reports document only total neurotransmitter contents. Considering the lack of vGAT and synaptic release during early development and the high intracellular taurine concentration, the abundance of taurine in the interstitial space may be considerably higher than that of GABA. On the other hand, the amount of taurine released by CP neurons is similar to the GABA release (Behar et al., 2001), indicating that at least in this niche GABA will by its substantially higher affinity to both GABA_A_ and GABA_B_ receptors mediate a more pronounced effect.

Overall, the observations summarized in this review indicate (i) that the molecular constituents of the GABAergic system are present at very early developmental stages before the onset of synaptogenesis, (ii) that tonic GABAergic currents are acting in the developing CNS, (iii) that GABA mediates a trophic action on neurogenesis, neuronal migration and differentiation in developmental niches which lack synaptic GABAergic signaling, and (iv) that tonic GABAergic currents regulate neuronal activity even after the establishment of reliable GABAergic synaptic transmission. In summary, all these studies provide compelling evidence for the important role of extrasynaptic GABAergic signaling during early neuronal development. Accordingly, it has been found that substances, which interfere with the GABAergic system during such early developmental stages, and thus affect mostly non-synaptic processes, disturb the proper development of the central nervous system. For example, antiepileptic drugs acting on GABA mechanisms lead to hippocampal and neocortical dysplasias, most probably by disturbing proliferation and radial migration (Manent et al., 2007). Similarly, prenatal exposure to ethanol, which is supposed to act partially via an activation of δ subunit containing receptors (Brickley and Mody, 2012), leads to a decreased proliferation (Jacobs and Miller, 2001) and migration (Cuzon et al., 2008) of cortical neurons. These examples demonstrate that the important role of extrasynaptic GABAergic signaling during neuronal development has to be considered in therapeutical intervention during pregnancy. On the other hand, the early expression of different elements of the GABAergic system during very early stages of neuronal development emphasizes the close interaction between genetic programs and functional responses of neuronal progenitors or neuroblasts. To elucidate how genetic programs determine the electrical properties of the developing nervous system and how, vice versa, the tonic activation of neurotransmitter receptors influence the transcription of genes will be essential questions for the further comprehension of early neuronal development.

## Conflict of Interest Statement

The authors declare that the research was conducted in the absence of any commercial or financial relationships that could be construed as a potential conflict of interest.
